# Domestic violence laws and women’s unmet need for family planning: Quasi-experimental evidence from Africa

**DOI:** 10.1186/s12978-025-02011-3

**Published:** 2025-04-26

**Authors:** Pragya Bhuwania, Amy Raub, Aleta Sprague, Alfredo Martin, Bijetri Bose, Rachel Kidman, Jody Heymann

**Affiliations:** 1 WORLD Policy Analysis Center, Fielding School of Public Health, University of California, 621 Charles E Young Drive South, Los Angeles, CA 2225 LSB90095 USA; 2 Department of Family, Population and Preventive Medicine, Stony Brook University, Stony Brook, NY 11794-8338 USA

**Keywords:** Women’s unmet need for family planning, Laws against domestic violence, Intimate partner violence, Reproductive health rights, Africa, Effectiveness

## Abstract

**Background:**

Approximately 164 million women report an unmet need for family planning globally. This has far-reaching consequences for the health of women and their children. Women’s exposure to intimate partner violence (IPV) is strongly linked to increased unmet need as IPV likely affects both women’s desire for contraception and their ability to access it. Around 245 million women were subject to physical and/or sexual IPV by an intimate partner in the past twelve months alone, making it the most common form of violence against women. Yet, laws that prohibit domestic violence (DV) are not universal and countries actively debate whether legal provisions are effective in deterring and reducing its harmful impacts. This study examines the impact of DV laws on women’s unmet need for family planning.

**Methods:**

We built new data on DV laws adoption in Africa and used the Demographic and Health Surveys (DHS) data collected between 2000 and 2021 across 23 African countries for outcomes data. Exploiting the staggered adoption of DV laws across the continent, we used a difference-in-differences study design to estimate the impact of DV laws in the treated countries compared to countries without such laws.

**Results:**

We find that DV laws reduced women’s unmet need for family planning by 6.2% points, 95% CI [− 9.2, − 3.2], a 20.5% reduction from the mean. Positive impacts were observed in 6 countries across multiple model specifications. While the impacts were largely broad-based across wealth, age, geography, and education categories, we found no significant impacts for women younger than 20 years of age and those without formal education. These findings were robust to alternative model specifications.

**Conclusions:**

Our findings demonstrate that countries can significantly benefit from prohibiting DV as a fundamental step towards addressing women’s unmet need for family planning and promoting their reproductive health. While fully addressing IPV and women's unmet need may require a range of complementary interventions, especially among marginalized populations, DV laws play a crucial role in improving women's control over their reproductive health.

**Supplementary Information:**

The online version contains supplementary material available at 10.1186/s12978-025-02011-3.

## Background

Freedom from violence and the full realization of reproductive rights are both essential to women’s bodily autonomy, health, and opportunities. Yet the world has far to go in achieving either. According to 2018 WHO global estimates, around 245 million women were subject to physical and/or sexual intimate partner violence (IPV) annually, which remains the most common form of violence against women [1]. Meanwhile, approximately 164 million women report an unmet need for contraception [2]. The consequences of IPV for individual women as well as their children, broader communities, and countries are profound.

IPV can result in extensive and debilitating harm to physical and mental health, with specific consequences including injury, stress-related gastrointestinal disorders, sexually transmitted infections, gynecological problems, chronic pain, depression, anxiety, insomnia, post-traumatic stress disorder, and death, among others [3, 4]. Women experiencing IPV may also face economic obstacles to accessing health care, including barriers to working or lose their jobs as a result of the violence, making it more difficult for them to leave a violent relationship or attain economic security and independence long-term [5]. IPV also has grave intergenerational health consequences, with children’s exposure to IPV associated with higher risks of neurological disorders, chronic stress, sleep problems, frequent headaches, asthma, emotional and behavioral problems, post-traumatic stress and anxiety disorders, poor nutrition outcomes, as well as education disruptions, substance use disorders, and barriers to employment in adulthood [6, 7]. Given its collective toll on health, work, and livelihoods, UN Women has estimated that IPV costs the global economy around $1.5 trillion per year [8].

Among the many consequences of IPV, studies across the globe have found that IPV is associated with adverse reproductive health outcomes, including unmet need for family planning [9–11] and unintended pregnancies [12–18]. Unmet need for contraception is widely recognized as a barrier to women’s reproductive health rights to freely choose the number, timing, spacing, and partners with whom to have children [19]. Unintended pregnancies have profound health implications for mothers and their children. Where reproductive healthcare is limited and abortion laws are restrictive, women facing unintended pregnancies often resort to unsafe abortion methods. Studies have found that complications due to unsafe abortions account for 8% to as many as 13% of maternal deaths each year [20]. Women with unintended pregnancies are vulnerable to increased rates of morbidity and mortality, often exacerbated by poverty, malnutrition, and limited healthcare access [21, 22]. Children born from unintended pregnancies are found to have an elevated risk of infant and child mortality [23–25]. Closely spaced pregnancies lead to complications like low birth weight, premature birth, and small for gestational age [26].

**Fig. 1:**
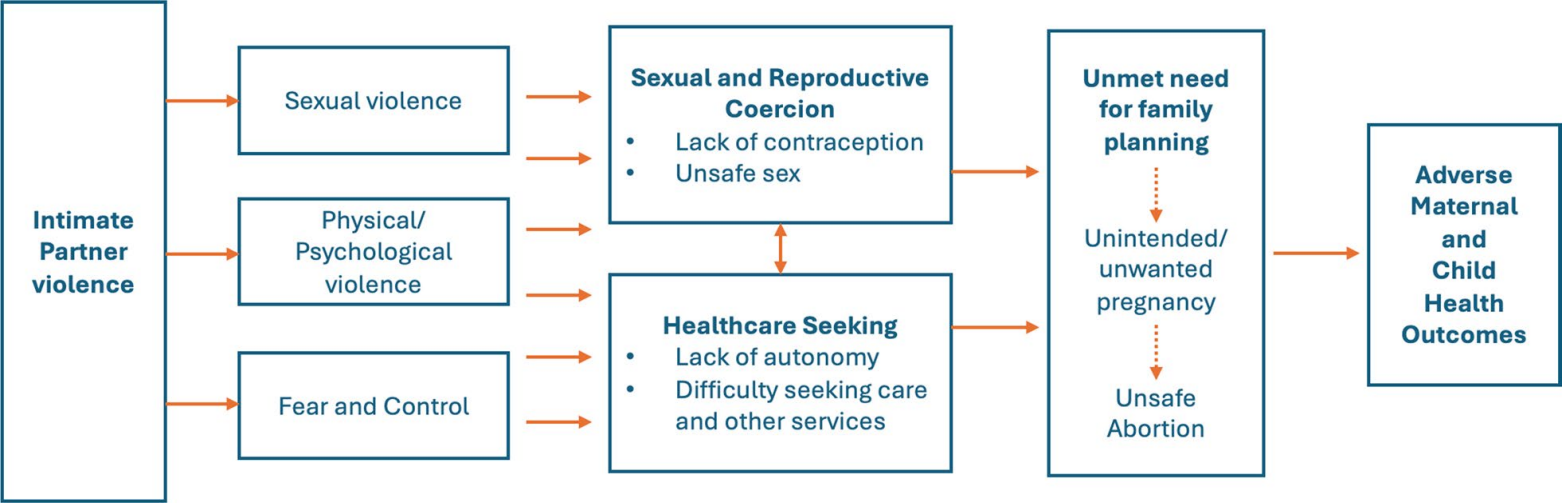
Pathways for IPV’s impact on women’s unmet need for family planning. Modified from García-Moreno et al. [31], Moore et al. [29], and Coker [32]

Married or cohabiting women rarely cite lack of access or cost related barriers as reasons for not using contraception and DHS data have been useful in revealing reasons why women may not seek contraception [27, 28]. A study of DHS data across 52 countries from 2005 to 2014 found that approximately 23% women were not using contraception despite wanting to avoid pregnancy because they or others close to them opposed contraception [27]. Women’s exposure to IPV is strongly linked to increased unmet need for family planning through multiple pathways (see Fig. 1). First, sexual violence, a common manifestation of IPV, directly infringes upon women's reproductive rights, resulting in forced or unprotected sex [29]. Second, abusive men are more likely to engage in contraceptive manipulation and contraceptive sabotage to gain control over their partner’s use of family planning and increase their partner’s dependency [30]. Finally, violent relationships are often characterized by fear and controlling behaviors by partners, undermining women’s ability to negotiate contraceptive use for fear of violence [29, 31].

Because IPV is likely to impact both women’s desire for contraception and their ability to access it [33, 34], interventions that address IPV may also have benefits for women’s unmet need for family planning. Yet few causal studies have examined the impact of domestic violence legislations [35, 36], and no causal studies have looked at what works at a national scale to address IPV and its impact on unmet need for contraception in LMICs. Domestic violence legislations and other laws advancing gender equality can both directly support the realization of women’s fundamental rights by deterring discriminatory acts and providing new mechanisms for recourse when rights are violated and can also meaningfully advance gender-equitable norms, which is critical to long-term change [37, 38]. At the same time, legal remedies may be out of reach for the most marginalized women and ensuring that domestic violence laws in particular are both effectively and equitably enforced has been a longstanding challenge across countries. Stigma and family or cultural pressure to avoid reporting, geographic and socioeconomic barriers to courts and lawyers, and inadequate budgets for investigating and prosecuting IPV claims may all hinder effective implementation, especially by women with fewer economic resources. Results of associational studies have been mixed [39, 40].

This study helps to fill these gaps in the literature by using longitudinal data to examine whether national laws prohibiting domestic violence affect rates of unmet need for contraception. This study focuses on Africa where high rates of IPV have been reported [1] and unmet need for contraception remains high despite substantial progress over the past twenty years [2]. There is also substantial variation within the region in IPV prevalence and contraception use [2, 41, 42]. Associational studies across African countries have also found a relationship between exposure to sexual violence, unmet need, and contraception usage [9, 43–45], as well as unwanted pregnancies [46, 47]. By rigorously comparing women’s experiences in countries that enacted domestic violence laws with those that did not, this study offers causal evidence into the relationship between IPV and unmet need for family planning and how these impacts vary among women with different socioeconomic backgrounds, while generating actionable evidence about the potential of national laws to help address these dual major challenges.

## Methods

We use difference-in-differences (DD), a widely used quasi-experimental research design, to estimate the impact of domestic violence laws on women’s unmet need for family planning. Exploiting the natural variation in the timing of policy adoption across countries, the DD research design identifies policy impact by comparing the trends in outcomes within and across treatment and comparison countries over the study period. Beginning in the early 2000s, many countries across Africa adopted laws prohibiting domestic violence while many others are yet to legally protect women against domestic violence. We use this difference in legal provisions against domestic violence across countries to isolate the impact of domestic violence laws on unmet need from overall trends across the continent.

### Policy and outcomes data

The policy data comes from the longitudinal database on domestic violence (DV) laws created by the authors and the WORLD Policy Analysis Center at UCLA. The database covers the period 1990–2021 and uses rigorous techniques developed by WORLD to translate original legislative text into quantitative measures [48], including independent double coding by two policy analysts for each country. This coding process captured how each country's laws regarding domestic violence changed over time, focusing on three key aspects: (i) existence of a specific law against domestic violence, (ii) availability of restraining orders as a legal protection measure, and (iii) classification of domestic violence as a criminal offense. Countries with legislations specifically addressing domestic violence, broader laws prohibiting violence against women, or amendments to penal codes that criminalized domestic violence and established penalties were coded as having legal protections against DV starting from the year the law came into force. Countries with no laws against domestic violence or those lacking mechanisms for victims to seek recourse, such as criminal penalties or restraining orders, were coded as having no legal protections throughout the study period. Notably, no additional or gradual changes in DV laws were observed in the treatment countries during the study period.

The outcomes data came from the Demographic and Health Surveys (DHS) harmonized by the Integrated Public Use Microdata Series (IPUMS). In cases where a harmonized version wasn't available, a researcher at our center harmonized the data from the original DHS survey following the IPUMS codebook. This approach ensured all surveys used the same methods. The DHS surveys target women aged 15–49 and gather in-depth information on health and demographics across over 90 low- and middle-income countries at regular intervals. The repeated cross-sectional DHS data are ideal for making comparisons across many countries over time due to the standardized way these surveys are conducted and their consistent measurement techniques. For our analysis, we specifically focused on African countries with at least two DHS surveys containing family planning data collected between 2000 and 2021. We focused on all married or cohabiting women born after 1975. These restrictions allowed us to examine changes in unmet need for family planning within each country over time.

We merged the domestic violence legislative database and the outcomes data collected by the DHS for countries that met our inclusion criteria in Africa. This results in an analytical sample pooled data from 69 DHS surveys across 23 countries (see Table 1). We dropped surveys conducted within two years of DV laws’ adoption because the time elapsed would have been insufficient for implementation and to determine treatment effects. The treatment group comprised 11 countries that have data on women surveyed before and after the adoption of DV laws. The comparison group included 12 countries that either lacked domestic violence laws entirely or implemented them after the most recent available national survey. We used only pre-reform data in Nigeria and included them as part of the comparison group because Nigeria’s DV law was not national.

**Table 1:** Treatment and comparison countries as per changes in domestic violence laws and DHS survey availability

Country	DHS	DV Policy
Treatment countries (11)		
Benin	2001, 2006, 2011, 2017	2012
Ethiopia	2000, 2011, 2016	2005
Ghana	2003, 2014	2007
Lesotho	2004, 2009, 2014	2010
Madagascar	2003, 2008, 2021	2005
Malawi	2000, 2004, 2010, 2016	2006
Namibia	2000, 2006, 2013	2003
Rwanda	2000, 2005, 2010, 2014, 2019	2008
Uganda	2001, 2006, 2016	2010
Zambia	2001, 2007, 2014, 2018	2011
Zimbabwe	2005, 2010, 2015	2006
Comparison countries (12)		
Burkina Faso	2003, 2010	2018
Chad	2004, 2014	2017
Guinea	2005, 2012	2016
Kenya	2003, 2008, 2014	2015
Liberia	2007, 2013	2018
Nigeria*	2003, 2008, 2013	2015
Cameroon	2004, 2011, 2018	NA
DRC	2007, 2013	NA
Egypt	2000, 2005, 2008, 2014	NA
Mali	2001, 2006, 2012, 2018	NA
Niger	2006, 2012	NA
Tanzania	2004, 2010, 2015	NA

*Note*: Excluded surveys conducted within two years of policy change

*We only included surveys that were conducted before the adoption of DV law in Nigeria and included them as part of the comparison group because Nigeria’s DV law was not national

### Measures

#### Outcome variable

We used a dichotomous variable indicating women’s unmet need for family planning (yes/no) using DHS’s revised definition of unmet need [49]. It is the sum of unmet need for spacing (women who wanted to delay having another child) and limiting (women who did not want another child) among women of reproductive age who were married or cohabiting, fecund, and/or sexually active. We constructed a binary variable (see Additional file 1 for details) using this information by coding all women reporting an unmet need for spacing or limiting as 1 and all other women who reported using a contraceptive, having no unmet need, or having a desire for birth within the next 2 years as 0. We excluded women who were infecund, menopausal, or sexually inactive because they had no need for family planning. This is a standard definition of unmet need used across studies in Africa using DHS data [9, 50, 51].

### Primary independent variable: Exposure to DV laws

We considered women legally protected against domestic violence if they were surveyed after DV laws were in force. The legal protections provided women with access to recourse either in the form of restraining orders or criminal penalties against the perpetrator. All women who were surveyed before countries passed DV laws constituted the unexposed category. Table 2 shows the sample sizes and the prevalence of unmet need for family planning among married or cohabiting women in exposed and unexposed categories for each country in the sample. Among a total of 367,365 married or cohabiting women of reproductive age, 123,199 women were exposed to DV laws. In the treatment group, unmet need was 30.3% among unexposed women and 21.3% among women exposed to DV laws. Unmet need was 22.4% in the comparison group over the study period.

**Table 2:** Sample size and sample characteristics of women currently married or living with a partner in the treated and comparison countries

Country	DV Law		Unmet need	
	Not exposed N	Exposed N	Not exposed % (N)	Exposed % (N)
Treated				
Benin	16,902	8933	31.4% (5307)	36.7% (3280)
Ethiopia	2856	15,165	34.5% (985)	22.4% (3404)
Ghana	1259	3678	40.6% (511)	31.9% (1173)
Lesotho	4383	2777	29.9% (1311)	18.1% (504)
Madagascar	1920	17,117	20.9% (401)	17.0% (2906)
Malawi	8392	23,967	30.4% (2555)	22.4% (5366)
Namibia	538	3548	28.3% (152)	20.1% (713)
Rwanda	3659	16,026	36.2% (1323)	15.4% (2471)
Uganda	4760	9462	36.0% (1715)	29.2% (2760)
Zambia	4540	13,664	25.7% (1169)	19.8% (2702)
Zimbabwe	2827	8862	11.5% (325)	10.3% (916)
All treated	52,036	123,199	30.3% (15,754)	21.3% (26,195)
Comparison				
Burkina Faso	12,742	–	25.5% (3249)	–
Cameroon	16,034	–	26.2% (4204)	–
Chad	12,381	–	25.4% (3141)	–
DRC	12,522	–	30.7% (3844)	–
Egypt	32,931	–	11.0% (3615)	–
Guinea	7181	–	24.4% (1755)	–
Kenya	11,771	–	22.2% (2617)	–
Liberia	5871	–	39.9% (2345)	–
Mali	22,229	–	28.7% (6384)	–
Niger	11,125	–	17.9% (1994)	–
Nigeria	33,971	–	19.6% (6652)	–
Tanzania	13,372	–	24.2% (3233)	–
All comparison	192,130	–	22.4% (43,033)	–

### Control variables

We included as control variables women- and household-level characteristics that are identified as key determinants of unmet need for family planning, particularly in Africa [51, 52]. Women’s characteristics included her age in 5-year intervals, total years of education, and age at first cohabitation. Household characteristics comprised total number of members in the household, wealth status in quintiles, and urban/rural location. We also included country-level urbanization rates over time to account for changes in access to family planning services over time.

### Statistical analysis

We used a two-way fixed effects (TWFE) model that allows for multiple groups to change over multiple periods in a difference-in-differences (DD) study setup. It is widely used to estimate the effect of laws and policies [53–55]. TWFE models estimate weighted average treatment effects across countries and time periods while controlling for country-specific and time-specific differences. Accordingly, we use the following equation:

1 $${Probit(Y}_{ict})={\beta }_{0}+{\beta }_{1} {DV\_law}_{ict}+ \delta {X}_{ict}+\xi {X}_{ct}+{\gamma }_{c}+ {\delta }_{t}+{e}_{ict}$$

to estimate the impact of DV laws on women’s unmet need for family planning. ${Y}_{ict}$ was a binary variable indicating whether woman *i* surveyed in country *c* in year *t* had an unmet need for family planning and ${DV\_law}_{ict}$ captured exposure to a DV law (it takes the value 1 for all women surveyed after the adoption of DV laws in treated countries and 0 otherwise). The main coefficient of interest was ${\beta }_{1}$ that estimates the effect of prohibiting domestic violence in treated countries compared to countries without DV laws. ${X}_{ict}$ represented individual- and household-level characteristics. The model accounted for time-invariant differences among countries as well as shared secular time trends over the study period across countries using country (${\gamma }_{c}$) and year (${\delta }_{t}$) fixed effects. Finally, ${X}_{ct}$ controlled for potential time-varying country-level confounders.

We used probit models because they are appropriate to analyze binary outcome variables. To estimate the output in terms of predicted probabilities, we calculated average marginal effects using the post-estimation commands, and report differences in the probabilities of unmet need. All models accounted for individual-level de-normalized sampling weights as recommended by the DHS for carrying-out multi-survey pooled analysis [56] and incorporated robust standard errors clustered at the country level.

Apart from estimating the pooled average effect of DV laws across all treatment countries, we also estimated the impact of DV laws in each treatment country individually. To examine country-specific impacts, we compared each treatment country at a time to the comparison group using the same estimation strategy. We could not carry out separate analysis for Benin and Uganda due to lack of overlapping data in the treatment and comparison groups in either the pre- or the post-reform period.

All statistical analyses were conducted using STATA 14.2. The study did not require IRB approval because it used publicly available data that are not individually identifiable.

### Model assumptions

The validity of TWFE DD estimates rests upon the parallel trends assumption that the trends in unmet need would have remained similar across the treatment and comparison groups in the absence of DV laws. We tested this assumption using the event-study model. The event-study design uses a set of dummy variables to capture the impact of an event in each time period. The data are centered at the time of policy adoption (t = 0) and a set of leads and lags comprise the periods before and after the event. We tested the parallel trends assumption by examining all coefficient estimates during the pre-reform period with reference to the last unexposed group of women (t =− 1). The post-reform coefficient estimates indicate how treatment effects vary over time after the adoption of DV laws.

### Additional analyses

While we looked at the overall impact of DV laws across all treatment countries and each one of them individually as well, the effects of these laws may vary across women depending on their socioeconomic backgrounds or other characteristics that determine access and use of family planning services. We report the heterogeneity of treatment effects across location, wealth, education, age, timing of marriage, and fertility preferences. Using linear probability models (LPM), we interacted policy exposure with each of these characteristics to determine differential policy effects. We report both the average effect of DV laws on each subcategory as well as the differential effect of the subcategories with respect to the reference omitted category to test statistical significance.

We conduct a range of sensitivity analyses to test the robustness of our findings. All models presented control for individual-level covariates and country-level urbanization over time. While religious beliefs may impact women’s unmet need, we do not include religion as a covariate in the main analysis due to data quality and availability issues. Similarly, we do not include partner’s education in the main analysis due to sample attrition. We also consider two macroeconomic indicators that, while not perfect, indicate access to and affordability of family planning services in a country. GDP per capita growth indicates the overall economic situation but could potentially be endogenous if DV laws enable more women to join the labor force and increase their incomes. Domestic governmental health expenditure (as a % of GDP) indicates public investments in overall healthcare, not limiting to family planning services alone. We present analyses including these variables as sensitivity checks.

Additionally, the main pooled analyses comprise 11 treatment countries including Benin and Uganda that were not individually estimable due to lack of overlapping data with the comparison countries. We dropped these countries from the pooled sample to test the sensitivity of the pooled treatment effects to exclusion of countries with insufficient data for country-specific analysis.

TWFE DD models are an extension of the simple 2-group × 2-time period DD models wherein the coefficient is a weighted average of treatment effects across all groups and times (Goodman-Bacon 2018) [57, 58]. Sometimes when the treatment timing varies across groups, negative weights may occur if already-treated units act as controls and changes in their treatment effects over time get subtracted from the DD estimate. We examined whether negative weights arise in our analysis and whether the treatment effects are sensitive to these occurrences.

We also re-ran the pooled analysis using the logit model that is widely used to study dichotomous outcome variables. Lastly, as a final sensitivity check, we estimated the model on a subset of currently married or cohabiting women who report no desire for births within the next two years i.e. women who indicate a demand for family planning. Because demand for FP may itself be susceptible to sample selection bias, the main results follow the standard practice of unmet need expressed as a percentage based on women who are married or in a union.

## Results

### Effects of domestic violence laws on women’s unmet need

Table 3 reports the marginal effects (difference in predicted probabilities in absolute percentage points) from the probit TWFE model shown in Eq. (1). These estimates measure the impact of prohibiting domestic violence on the probabilities of unmet need for family planning when compared to countries without such laws. Column (1) shows the estimates from the null model with only country and year fixed effects. Column (2) builds on the null model by controlling for individual-level confounders and finally, column (3) additionally controls for country-level urbanization levels that varies over time. We find that prohibiting domestic violence reduced women’s unmet need for family planning across all specifications. DV laws reduced the probability of unmet need by 6.2 percentage points overall, a 20.5% reduction from the mean.

**Table 3:** Impact on unmet need for family planning among married or cohabiting women of reproductive age

Country	Sample size (% exposed)	Unmet need treatment effects^†^ (95% CI)	Unmet need treatment effects^†^ (95% CI)	Unmet need treatment effects^†^ (95% CI)
		(1) Null model	(2) Individual-level covariates	(3) Individual and country-level covariates
**Pooled**	367,208	− 5.7***	− 6.0***	− 6.2***
	(33.6%)	(− 8.4, − 3.0)	(− 8.8, − 3.2)	(− 9.2, − 3.2)
Ethiopia	176,082	− 11.6***	− 13.4***	− 14.9***
	(8.6%)	(− 13.7, − 9.6)	(− 15.3, − 11.4)	(− 17.3, − 12.4)
Ghana	162,998	− 0.75	− 0.25	11.04
	(2.3%)	(− 6.1, 4.6)	(− 6.1, 5.6)	(− 6.3, 28.4)
Lesotho	165,219	− 10.6***	− 10.0***	− 3.4
	(1.7%)	(− 13.1, − 8.2)	(− 12.5, − 7.4)	(− 13.5, 6.7)
Madagascar	177,097	0.17	0.17	0.4
	(9.7%)	(− 2.4, 2.8)	(− 2.6, 2.9)	(− 1.2, 1.9)
Malawi	190,420	− 2.8***	− 3.7***	− 8.7***
	(12.6%)	(− 4.7, − 0.9)	(− 5.9, − 1.4)	(− 14.3, − 3.0)
Namibia	162,143	− 10.1***	− 10.3***	− 1.4
	(2.2%)	(− 11.4, − 8.9)	(− 11.9, − 8.7)	(− 15.2, 12.3)
Rwanda	177,739	− 11.6***	− 12.2***	− 12.4***
	(9.0%)	(− 12.8, − 10.4)	(− 13.5, − 10.9)	(− 13.1, − 11.8)
Zambia	176,250	− 3.7***	− 3.8***	− 6.5***
	(7.8%)	(− 4.2, − 3.2)	(− 4.5, − 3.1)	(− 9.5, − 3.6)
Zimbabwe	169,750	1.3	0.4	− 4.8
	(5.2%)	(− 1.5, 4.1)	(− 2.5, 3.2)	(− 16.4, 6.8)

^†^Treatment effects reported the change in the probability of unmet need in percentage points terms with 95% confidence intervals (CI)

*** *p* < 0.01, ** *p* < 0.05, * *p* < 0.1. Standard errors clustered at the country-level

TWFE model with survey year fixed effects and country fixed effects

Controls: Individual-level controls include women’s age in 5-year intervals, education, age at first marriage, family size, wealth, and rural/urban residence. Country-level controls include country’s urbanization rates over time

Table 3 also reports the impact of domestic violence laws on each of the treatment countries individually. We find consistent declines in 4 countries—Ethiopia, Malawi, Rwanda, and Zambia. Ethiopia witnessed greatest reduction of 14.9 percentage points, followed closely by Rwanda with 12.4 percentage points due to passage of domestic violence laws. The probability of unmet need declined by 8.7 percentage points in Malawi and by 6.5 percentage points in Zambia. Lesotho and Namibia show reductions in unmet need by around 10 percentage points across the null model and the model controlling for individual-level characteristics. These impacts are sensitive to the inclusion of country-level urbanization rates. Ghana, Madagascar, and Zimbabwe do not show any impact of domestic violence laws on women’s unmet need for family planning. The statistically non-significant results may also be due to insufficient sample sizes before or after policy implementation in many of these countries.

### Examining model assumptions

**Fig. 2:**
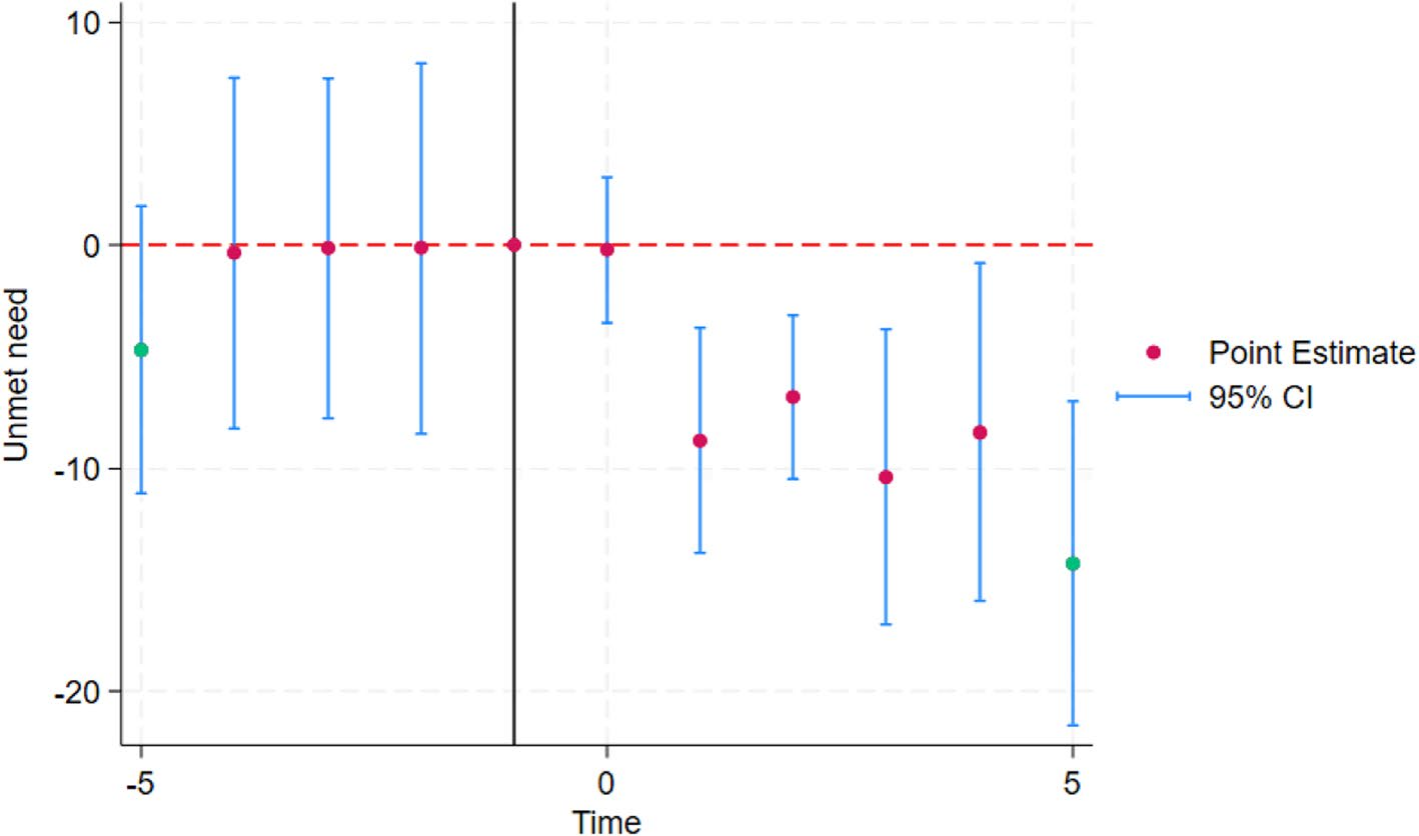
Event-study estimates of the impact of DV laws on the probability of unmet need. *Notes*: Each time point represents two-year intervals to improve estimation. The graph shows the difference in the probability of unmet need between treatment and comparison countries. Time is centered at the last untreated group. Point estimates of the lag and lead terms are presented along with their 95% confidence intervals. The omitted base category is taken as 1 year prior to the reform, indicated by the solid vertical line. The pre-reform period shows no evidence of divergent pre-trends, and the post-reform period is suggestive of homogenous treatment effects over time

The fundamental model assumption is that the trends in unmet need would have been similar across treatment and comparison countries in the absence of DV laws. To test parallel trends before the adoption of DV laws and homogenous treatment effects over time, we present the event-study plot for the pooled analysis [59] in Fig. 2. Figure 2 provides evidence that there were no differential trends in the probability of unmet need between treatment and comparison countries before the adoption of DV laws. It also indicates homogenous treatment effects over time except a slightly greater impact during the last time interval.

In addition to the parallel trends assumption, another significant challenge to the validity of DD estimates concerns their ability to isolate the impact of the policy from other concurrent changes that might have occurred during the study period. To test the possibility of other concurrent changes impacting access to family planning, we run the model on a group of women who would not be expected to be impacted by DV laws: women who were not married or living with a partner at the time of survey. If the observed change among married or cohabiting women is solely due to DV laws, then the placebo group shouldn’t show any significant change because DV laws do not apply to this group. We find that DV laws had no impact (1.7, 95% CI [− 9.2, 12.6]) on the unmet need for family planning among these women. These findings strengthen the argument that the decline in unmet need followed from the passage of DV laws and not any other policy changes (such as those increasing access to family planning services) or other macroeconomic factors that would have impacted the placebo group as well.

### Heterogeneity analyses

In Table 4, we report the heterogeneity of treatment effects across location, wealth, education, age, timing of marriage, and fertility preferences. In Panel A, we find that DV laws impacted women across urban and rural areas. While the impact was substantially higher among women residing in urban areas at 10.4 percentage points than those residing in rural areas at 6.7 percentage points, the difference was not statistically significant. Panel B shows that the magnitude of the impact of DV laws increased significantly with women’s wealth background. The impact was the lowest among the poorest women at 4.5 percentage points and the highest among the richest women at 11.4 percentage points. Panel C highlights the vulnerability of women without any education as opposed to women with at least primary education. DV laws had no impact on women who were not educated whereas the impact grew with women’s education level from 8.0 percentage points for women with primary education to 11.6 percentage points for women with higher education. All differences were statistically significant when compared to women with no education.

**Table 4:** Heterogeneity in the impact of DV laws on women’s unmet need for family planning across different groups of women

Country	Unmet need treatment effects^a^ (95% CI)	Unmet need interaction effects^b^ (95% CI)
A: Location		
Rural (base)	− 6.7*** (− 10.3, − 3.1)	
Urban	− 10.4*** (− 16.4, − 4.4)	− 3.7 (− 9.5, 1.9)
B: Wealth		
Poorest (base)	− 4.5** (− 8.7, − 0.3)	
Poor	− 5.2*** (− 8.8, − 1.5)	− 0.7 (− 3.1, 1.7)
Middle	− 7.8*** (− 11.5, − 4.2)	− 3.3* (− 7.3, 0.6)
Rich	− 9.5*** (− 13.8, − 5.3)	− 5.0** (− 10.1, − 0.0)
Richest	− 11.4*** (− 17.5, − 5.3)	− 6.9** (− 13.1, − 0.7)
C: Education		
No education (base)	− 3.4 (− 7.8, 1.1)	
Primary	− 8.0*** (− 11.5, − 4.4)	− 4.6*** (− 7.5, − 1.7)
Secondary	− 9.4*** (− 14.5, − 4.3)	− 6.0** (− 11.4, − 0.8)
Higher	− 11.6*** (− 18.7, − 4.4)	− 8.2*** (− 13.8, − 2.6)
D: Age		
15–19 (base)	− 2.8 (− 6.5, 0.9)	
20*–*24	− 9.5*** (− 13.8, − 5.4)	− 6.7*** (− 8.8, − 4.7)
25*–*29	− 8.7*** (− 12.4, − 5.1)	− 5.9*** (− 7.6, − 4.2)
30*–*34	− 7.6*** (− 11.3, − 3.9)	− 4.8*** (− 6.5, − 3.1)
35–39	− 5.6*** (− 9.2, − 2.2)	− 2.8*** (− 4.7, − 1.0)
40*–*45	− 11.2*** (− 18.7, − 3.7)	− 8.4** (− 14.8, − 2.0)
E: Marriage after DV law		
Before (base)	− 7.4*** (− 10.7, − 4.1)	
After	− 8.7*** (− 12.9, − 4.5)	− 1.3 (− 3.3, 0.6)
F: Partner’s fertility preferences^c^		
More (base)	− 6.1*** (− 10.4, − 1.8)	
Same	− 9.5*** (− 13.8, − 5.3)	− 3.4 (− 7.7, 0.9)
Fewer	− 8.5** (− 12.4, − 4.7)	− 2.4* (− 5.3, 0.4)

^a^Treatment effects reported the change in the probability of unmet need in percentage points terms with 95% confidence intervals (CI)

^b^Interaction effects show the additional change in the probability of unmet need for the subgroup with respect to the omitted base category with 95% CI

^c^Excluded women who reported they don’t know their partner’s fertility preferences

*** *p* < 0.01, ** *p* < 0.05, * *p* < 0.1

Standard errors clustered at the country-level

TWFE model with survey year fixed effects and country fixed effects

Controls: Individual-level controls include women’s age in 5-year intervals, education, age at first marriage, family size, wealth, and rural/urban residence. Country-level controls include country’s urbanization rates over time

Similarly, Panel D highlights the vulnerability of another group of women who are under 20 years of age. While DV laws had significant effects on women across different age groups ages 20 and older, they had no impact on women aged 15–19 years. In Panel E, we examine whether the impact varied across women who married before the passage of DV laws compared to those who married afterwards. The results show that DV laws impacted women regardless of whether they got married before or after the adoption of DV laws. Lastly, the impact of DV laws may vary depending on the difference in fertility preferences between the respondent and the partner. While DV laws reduced unmet need for family planning across the board, the impact was higher among women whose partners wanted the same or fewer children at about 9.5.-8.5 percentage points compared to the impact of 6.1 percentage points among women whose partners wanted more children than them.

### Sensitivity analyses

Table 5 presents a range of sensitivity analyses that we conducted to test the robustness of our findings. In Panels A and B, we see that controlling for partner’s level of education or woman’s religious beliefs in addition to previously included individual and country-level covariates does not change our results. Panel C shows that our main findings are also not sensitive to controlling for overall macroeconomic indicators such as GDP per capita growth and domestic government health expenditures. Panel D excludes countries with insufficient sample sizes for individual country-specific impact estimation. The pooled treatment effects remain stable.

**Table 5:** Sensitivity analyses to test the robustness of the impact of DV laws on women’s unmet need for family planning

Country	Sample size	Unmet need treatment effects^†^ (95% CI)
Panel A (*partner’s education*)	357,538	− 6.2*** (− 9.2, − 3.2)
Panel B (*religion*)	345,342	− 6.2*** (− 9.5, − 3.0)
Panel C (*macroeconomic characteristics*)	367,208	− 6.0*** (− 8.8, − 3.2)
Panel D (*dropping countries not individually estimable*)	327,158	− 6.1*** (− 9.2, − 2.9)
Panel E (*dropping country × years with negative weights*)	345,887	− 6.2*** (− 9.1, − 3.3)
Panel F (*logit model*)	367,208	− 5.9*** (− 9.0, − 2.9)
Panel G (*among women with a demand for FP*)	207,630	− 11.6*** (− 19.7, − 3.4)

^†^Treatment effects reported the change in the probability of unmet need in percentage points terms with 95% confidence intervals (CI)

*** *p* < 0.01, ** *p* < 0.05, * *p* < 0.1

Standard errors clustered at the country-level

TWFE model with survey year fixed effects and country fixed effects

Controls: Individual-level controls include women’s age in 5-year intervals, education, age at first marriage, family size, wealth, and rural/urban residence. Country-level controls include country’s urbanization rates over time. Macroeconomic characteristics in panel C include GDP per capita growth and domestic public health expenditure as a percentage of GDP

We examined whether negative weights arise in our analysis and tested the sensitivity of our treatment effects to these occurrences. We found only three country-year cases with negative weights (see Additional file 2). To assess their severity, we removed the data with negative weights from the analytical sample and re-ran the pooled analysis. The result in panel E is the same as our main result, suggesting that the presence of few negative weights did not impact the treatment effects. Lastly, Panel G shows that the coefficient estimate is larger in size among women with a demand for FP. DV laws reduced the overall unmet need by 11.6 percentage points, a 21.3% reduction from the mean, among women with a demand for FP.

## Discussion

This study uses a quasi-experimental design to evaluate the impact of national laws prohibiting domestic violence on women’s unmet need for family planning. We find that domestic violence laws reduced the probability of unmet need by 6.2 percentage points overall, a 20.5% reduction from the mean. As the first study using causal methods to measure how domestic violence laws affect unmet need for family planning, this research makes an important contribution to the literature. While prior research shows that experience of IPV is associated with higher probabilities of unmet need for family planning, our study demonstrates that laws prohibiting IPV have broader implications for the realization of women’s sexual and reproductive health and rights. These results are robust to different model specifications and assumption checks.

Despite the recognition that the effectiveness of DV laws can vary significantly depending on factors such as specific legal provisions, enforcement, support systems for survivors, and the broader socio-cultural context, this study found strong evidence that DV laws were associated with a reduction in women's unmet need for family planning across 11 countries in Africa. Underreporting and other obstacles may mean that the observed impact reflects partial implementation and likely represents a conservative estimate. Addressing these barriers, such as improving access to support services, enhancing survivor safety, and challenging societal norms that perpetuate IPV, could lead to a more significant and sustained reduction in IPV prevalence and its associated harms.

This observed reduction likely reflects a combination of normative shifts and law enforcement. Researchers have found that even weakly enforced DV laws can contribute to positive social change by transforming women’s attitudes toward violence and raising awareness about protecting their rights [38, 60]. DV laws may reduce the prevalence of IPV [35], which in turn reduces barriers to women meeting their family planning needs [9, 43]. Previous literature also demonstrated that DV laws improve women's household decision-making power regarding healthcare and finances [36], which is likely to affect women’s ability to access and use family planning.

Among the 11 treatment countries in our sample, the impacts of domestic violence laws on unmet need were greatest in Ethiopia (14.9 percentage points), Malawi (12.4 percentage points), Rwanda (8.7 percentage points), and Zambia (6.5 percentage points). No significant effects were found in Ghana, Madagascar, and Zimbabwe, but these non-significant results may reflect relatively small sample sizes. Differences in policy design aspects and quality of enforcement could help explain the variation in impacts across countries. Future studies should examine the impact of policy design decisions in DV laws, including what constitutes violence, who is covered, whether it’s a criminal offence and its associated penalties, and access to restraining orders and institutional support systems.

This study also provides new insights into whether domestic violence laws are reaching women equitably. Women across demographics experienced reductions in unmet need, though the magnitude of impact varied for women from different backgrounds. For example, impacts among the richest women were over twice as large as impacts among the poorest women, as measured by wealth quintiles. Similarly, women with higher education experienced a greater reduction in unmet need (11.6 percentage points) than women with only a primary education (8.0 percentage points). The only groups for whom we found no significant effects were women with no formal education and the youngest women (ages 15–19).

In demonstrating the role of domestic violence laws in reducing unmet need for contraception, this study highlights the benefits of laws to the urgent priorities of women’s health, autonomy, and fundamental rights. At the same time, it provides further evidence that the ability of women to decide freely about when, how, with whom, and how many children to have depends on their socioeconomic conditions, communities, and political surroundings. To fully realize reproductive justice, future research should examine whether knowledge of laws, implementation barriers, or other obstacles are driving the variation in these impacts across socioeconomic status and age, among other demographic characteristics. Given the justice system may have limitations [61] in adequately addressing this issue for the most vulnerable and marginalized women, it is critical to identify strategies for more effectively reaching those least served by DV laws.

Importantly, we recognize that prohibiting domestic violence is only one aspect of addressing women’s unmet need for family planning and is only one part of a comprehensive approach to addressing IPV. Future research should consider other factors such as the supply and ease of access to contraceptives and accurate knowledge and awareness of family planning services which are also critical to reducing women’s unmet need for family planning. Furthermore, addressing the vulnerability to IPV and the need for family planning services among unmarried or non-cohabiting girls and women requires further attention.

A common limitation of studies using the DD approach is that despite effectively controlling for secular time trends and fixed country-level characteristics, it's still possible that impact estimates capture the combined effect of the policy and unobserved changes happening within countries over time. We addressed this concern by presenting the results for the placebo group of women who were not married or in union. Our results show that DV laws did not impact unmet need among these women, while they did reduce unmet need among women who were married or cohabiting. The lack of a similar reduction in the placebo group suggests that the relationship between DV laws and unmet need is unlikely to be confounded by other coinciding changes that are likely to impact all women regardless of marital status. If other unobserved factors, like family planning services or campaigns, were significantly influential, we'd expect some effect in the placebo group, unless those factors exclusively targeted married women. We also show in the main results and sensitivity checks that these impacts are robust to the inclusion of other relevant time-varying country-level characteristics such as urbanization levels, GDP per capita growth rate, and domestic government health expenditures.

The study reports the average effect of these laws on women's unmet need across all countries in the sample. It also reports the impact in each country individually. However, the study design does not consider the varying durations of historical exposure to these laws across countries. Although the event-study plot (Fig. 2) suggests that, on average, the treatment effects remain consistent over time, variations in the duration of exposure to these laws across countries may explain the observed differences in their impact. Additionally, we used the most widely available and used definition of women’s unmet need available in the DHS surveys. However, several studies point to its limitations and have proposed new measurements that consider women’s fertility intentions and agency in different ways [62–64]. Future research examining how public policies affect different measures of women’s unmet need may provide further insights into the mechanism affected.

Another potential limitation is the presence of negative weights in the Two-Way Fixed Effects (TWFE) model [65]. However, the robustness checks we conducted suggest these negative weights do not have a significant impact on the overall findings. Additionally, the country-specific analyses avoid potential biases that can arise when previously treated countries are included as comparisons because we only used one treatment country at a time to estimate these impacts [66].

## Conclusion

IPV and barriers to women’s reproductive autonomy are closely linked. This study demonstrates that domestic violence legislation matters to reducing unmet need for contraception. While fully addressing both IPV and unmet need for contraception will require a wide range of legal, policy, and community interventions, laws addressing domestic violence represent one fundamental step that all countries can take. The potential benefits for women’s health, autonomy, and opportunities—and the intergenerational and societal harms that will result if the current scale of IPV and unmet need for contraception persist—underscore the urgency of action.

## Supplementary Information

Additional file 1.

Additional file 2.

## Data Availability

The authors will make available the original policy data at www.worldpolicycenter.org. The study also used outcomes data that are available from IPUMS and DHS Programs at the following addresses: https://www.idhsdata.org/idhs/ and https://dhsprogram.com/Data/.

## Abbreviations

WHO: World Health Organization
IPV: Intimate Partner Violence
DD: Difference-in-differences
DV: Domestic Violence
DHS: Demographic and Health Surveys
IPUMS: Integrated Public Use Microdata Series
TWFE: Two-way Fixed Effects
CI: Confidence Intervals

## References

[1] World Health Organization. Violence against women prevalence estimates, 2018: global, regional and national prevalence estimates for intimate partner violence against women and global and regional prevalence estimates for non-partner sexual violence against women. World Health Organization; 2021.

[2] UNDESA. World Family Planning 2022; 2022. Available at https://www.un.org/development/desa/pd/sites/www.un.org.development.desa.pd/files/files/documents/2023/Feb/undesa_pd_2022_world-family-planning.pdf.

[3] Campbell JC. Health consequences of intimate partner violence. The lancet. 2002;359(9314):1331–6. 10.1016/S0140-6736(02)08336-8 11965295

[4] White SJ, Sin J, Sweeney A, Salisbury T, Wahlich C, Montesinos Guevara CM, Gillard S, Brett E, Allwright L, Iqbal N, Khan A. Global prevalence and mental health outcomes of intimate partner violence among women: a systematic review and meta-analysis. Trauma Violence Abuse. 2024;25(1):494–511. 36825800 10.1177/15248380231155529 PMC10666489

[5] MacGregor JC, Oliver CL, MacQuarrie BJ, Wathen CN. Intimate partner violence and work: a scoping review of published research. Trauma Violence Abuse. 2021;22(4):717–27. 31615345 10.1177/1524838019881746

[6] Artz S, Jackson MA, Rossiter KR, Nijdam-Jones A, Géczy I, Porteous S. A comprehensive review of the literature on the impact of exposure to intimate partner violence on children and youth.

[7] Barnett W, Nhapi R, Zar HJ, Halligan SL, Pellowski J, Donald KA, Stein DJ. Intimate partner violence and growth outcomes through infancy: a longitudinal investigation of multiple mediators in a South African birth cohort. Matern Child Nutr. 2022;18(1): e13281. 34734487 10.1111/mcn.13281 PMC8710113

[8] UN Women. Remarks by UN assistant secretary-general and deputy executive director of UN Women, Lakshmi Puri at the high-level discussion on the “economic cost of violence against women.”; 2016. https://www.unwomen.org/en/news/stories/2016/9/speech-by-lakshmi-puri-on-economic-costs-of-violence-against-women. Accessed 21 May 2024.

[9] Ahinkorah BO, Ameyaw EK, Seidu AA, Agbaglo E, Budu E, Mensah F, Adu C, Yaya S. Sexual violence and unmet need for contraception among married and cohabiting women in sub-Saharan Africa: Evidence from demographic and health surveys. PLoS ONE. 2020;15(11): e0240556. 33141830 10.1371/journal.pone.0240556 PMC7608905

[10] Gomez AM. Sexual violence as a predictor of unintended pregnancy, contraceptive use, and unmet need among female youth in Colombia. J Womens Health. 2011;20(9):1349–56. 10.1089/jwh.2010.2518 21740193

[11] Hoo KY, Lai SL. Intimate partner violence and unmet need for family planning in selected south Asian countries. Int J Social Determinants Health Health Serv. 2023;53(1):48–62. 10.1177/00207314221131218 36214188

[12] Ajayi AI, Ezegbe HC. Association between sexual violence and unintended pregnancy among adolescent girls and young women in South Africa. BMC Public Health. 2020;20(1):1370. 32894130 10.1186/s12889-020-09488-6 PMC7487533

[13] Kasonde ME, Bwalya BB, Nyirenda ET, Mapoma CC, Sikaluzwe M, Chimpinde K, Songolo GI. Association between sexual violence and unintended pregnancy among married women in Zambia. BMC Public Health. 2022;22(1):1491. 35927643 10.1186/s12889-022-13881-8 PMC9354302

[14] Pallitto CC, García-Moreno C, Jansen HA, Heise L, Ellsberg M, Watts C. Intimate partner violence, abortion, and unintended pregnancy: results from the WHO Multi-country Study on Women’s Health and Domestic Violence. Int J Gynecol Obstet. 2013;120(1):3–9. 10.1016/j.ijgo.2012.07.003 22959631

[15] Tenkorang EY. Intimate partner violence and the sexual and reproductive health outcomes of women in Ghana. Health Educ Behav. 2019;46(6):969–80. 31319724 10.1177/1090198119859420

[16] Tiruye TY, Harris ML, Chojenta C, Holliday E, Loxton D. Intimate partner violence against women in Ethiopia and its association with unintended pregnancy: a national cross-sectional survey. Int J Public Health. 2020;65:1657–67. 33048193 10.1007/s00038-020-01510-3

[17] Titilayo A, Ekundayo O, Olaoye-Oyesola O, Anuodo A. Sexual violence as a predictor of unwanted pregnancy: evidence from the 2013 Nigeria Demographic and Health Survey. Eur Sci J. 2017;13(20):140.

[18] Yaya S, Amouzou A, Uthman OA, Ekholuenetale M, Bishwajit G, Udenigwe O, Hudani A, Shah V. Prevalence and determinants of terminated and unintended pregnancies among married women: analysis of pooled cross-sectional surveys in Nigeria. BMJ Glob Health. 2018;3(2): e000707. 29713502 10.1136/bmjgh-2018-000707 PMC5922476

[19] UNFPA. Human rights-based approach to family planning: UNFPA support tool. New York: UNFPA; 2023.

[20] Say L, Chou D, Gemmill A, Tunçalp Ö, Moller AB, Daniels J, Gülmezoglu AM, Temmerman M, Alkema L. Global causes of maternal death: a WHO systematic analysis. Lancet Glob Health. 2014;2(6):e323–33. 25103301 10.1016/S2214-109X(14)70227-X

[21] Hajizadeh M, Nghiem S. Does unwanted pregnancy lead to adverse health and healthcare utilization for mother and child? Evidence from low-and middle-income countries. Int J Public Health. 2020;65:457–68. 32270238 10.1007/s00038-020-01358-7 PMC7275006

[22] Klima CS. Unintended pregnancy: consequences and solutions for a worldwide problem. J Nurse Midwifery. 1998;43(6):483–91. 9871381 10.1016/s0091-2182(98)00063-9

[23] Adedini SA, Odimegwu C, Imasiku EN, Ononokpono DN. Unmet need for family planning: implication for under-five mortality in Nigeria. J Health Popul Nutr. 2015;33(1):187. 25995735 PMC4438662

[24] Rana MJ, Goli S. The road from ICPD to SDGs: health returns of reducing the unmet need for family planning in India. Midwifery. 2021;1(103): 103107. 10.1016/j.midw.2021.103107 34358778

[25] Singh S, Darroch JE, Ashford LS, Vlassoff M. Adding It Up: The costs and Benefits of Investing in family Planning and maternal and new born health. Guttmacher Institute; 2009.

[26] Conde-Agudelo A, Rosas-Bermudez A, Castaño F, Norton MH. Effects of birth spacing on maternal, perinatal, infant, and child health: a systematic review of causal mechanisms. Stud Fam Plann. 2012;43(2):93–114. 23175949 10.1111/j.1728-4465.2012.00308.x

[27] Sedgh G, Ashford LS, Hussain R. Unmet need for contraception in developing countries: examining women’s reasons for not using a method; 2016

[28] Sedgh G, Hussain R. Reasons for contraceptive nonuse among women having unmet need for contraception in developing countries. Stud Fam Plann. 2014;45(2):151–69. 24931073 10.1111/j.1728-4465.2014.00382.x

[29] Moore AM, Frohwirth L, Miller E. Male reproductive control of women who have experienced intimate partner violence in the United States. Soc Sci Med. 2010;70(11):1737–44. 20359808 10.1016/j.socscimed.2010.02.009

[30] Maxwell L, Devries K, Zionts D, Alhusen JL, Campbell J. Estimating the effect of intimate partner violence on women’s use of contraception: a systematic review and meta-analysis. PLoS ONE. 2015;10(2): e0118234. 25693056 10.1371/journal.pone.0118234 PMC4334227

[31] García-Moreno C, Pallitto C, Devries K, Stöckl H, Watts C, Abrahams N. Global and regional estimates of violence against women: prevalence and health effects of intimate partner violence and non-partner sexual violence. World Health Organization; 2013.

[32] Coker AL. Does physical intimate partner violence affect sexual health? A systematic review. Trauma Violence Abuse. 2007;8(2):149–77. 17545572 10.1177/1524838007301162

[33] Adjiwanou V, N’Bouke A. Exploring the paradox of intimate partner violence and increased contraceptive use in sub-Saharan Africa. Stud Fam Plann. 2015;46(2):127–42. 26059986 10.1111/j.1728-4465.2015.00020.x

[34] Alio AP, Daley EM, Nana PN, Duan J, Salihu HM. Intimate partner violence and contraception use among women in Sub-Saharan Africa. Int J Gynecol Obstet. 2009;107(1):35–8. 10.1016/j.ijgo.2009.05.002 19481751

[35] Beleche T. Domestic violence laws and suicide in Mexico. Rev Econ Household. 2019;17(1):229–48.

[36] Bhuwania P, Raub A, Sprague A, Martin A, Bose B, Kidman R, Nandi A, Behrman JR, Heymann J. Impact of laws prohibiting domestic violence on wasting in early childhood. PLoS ONE. 2024;19(3): e0301224. 38547244 10.1371/journal.pone.0301224 PMC10977741

[37] García-Moreno C, Zimmerman C, Morris-Gehring A, Heise L, Amin A, Abrahams N, Montoya O, Bhate-Deosthali P, Kilonzo N, Watts C. Addressing violence against women: a call to action. The Lancet. 2015;385(9978):1685–95. 10.1016/S0140-6736(14)61830-4 25467579

[38] Xu D. The wording matters: Gender equality laws and women’s attitudes towards domestic violence in Africa. Soc Sci Med. 2024;1(345): 116668. 10.1016/j.socscimed.2024.116668 38382333

[39] Fan X, Loria MV. Intimate partner violence and contraceptive use in developing countries. Demogr Res. 2020;1(42):293–342.

[40] Maxwell L, Khan Z, Yount KM. Do laws promoting gender equity and freedom from violence benefit the most vulnerable? A multilevel analysis of women’s and adolescent girls’ experiences in 15 low-and-middle-income countries. Health Policy Plan. 2022;37(1):33–44. 34698857 10.1093/heapol/czab127 PMC8757492

[41] Ahinkorah BO, Dickson KS, Seidu AA. Women decision-making capacity and intimate partner violence among women in sub-Saharan Africa. Arch Public Health. 2018;76:1. 29423218 10.1186/s13690-018-0253-9 PMC5787915

[42] Nabaggala MS, Reddy T, Manda S. Effects of rural–urban residence and education on intimate partner violence among women in Sub-Saharan Africa: a meta-analysis of health survey data. BMC Womens Health. 2021;21:1–23. 33849492 10.1186/s12905-021-01286-5 PMC8045348

[43] Deyessa N, Argaw A. Intimate partner violence and unmet need for contraceptive use among Ethiopian women living in marital union. Ethiopian J Health Dev. 2018;32(3).

[44] Muluneh MD, Francis L, Agho K, Stulz V. The association of intimate partner violence and contraceptive use: a multi-country analysis of demographic and health surveys. Int J Equity Health. 2023;22(1):75. 37101283 10.1186/s12939-023-01884-9 PMC10134549

[45] Okigbo CC. Factors associated with unmet need for modern contraception in post-conflict Liberia. Afr J Reprod Health. 2014;18(2):58–67. 25022142

[46] Ahinkorah BO, Seidu AA, Appiah F, Oduro JK, Sambah F, Baatiema L, Budu E, Ameyaw EK. Effect of sexual violence on planned, mistimed and unwanted pregnancies among women of reproductive age in sub-Saharan Africa: a multi-country analysis of demographic and health surveys. SSM-Population Health. 2020;1(11): 100601. 10.1016/j.ssmph.2020.100601 PMC7276486 32529021

[47] Muluneh MD, Francis L, Agho K, Stulz V. The relationship between intimate partner violence and unintended pregnancy: Eastern Sub Saharan African countries’ analysis of demographic and health surveys. J Interpers Violence. 2023;38(7–8):5375–403. 36073139 10.1177/08862605221120894

[48] Raub A, Sprague A, Waisath W, Nandi A, Atabay E, Vincent I, Moreno G, Earle A, Perry N, Heymann J. Utilizing a comparative policy resource from the WORLD policy analysis center covering constitutional rights, laws, and policies across 193 countries for outcome analysis, monitoring, and accountability. J Comparative Policy Anal Res Practice. 2022;24(4):313–28.

[49] Bradley SE, Croft TN, Fishel JD, Westoff CF. Revising unmet need for family planning; 2012.

[50] Kabagenyi A, Wasswa R, Kayemba V. Multilevel mixed effects analysis of individual and community factors associated with unmet need for contraception among married women in four East African countries. SSM-Population Health. 2024;1(25): 101602. 10.1016/j.ssmph.2024.101602 PMC10875291 38375320

[51] Teshale AB. Factors associated with unmet need for family planning in sub-Saharan Africa: a multilevel multinomial logistic regression analysis. PLoS ONE. 2022;17(2): e0263885. 35143584 10.1371/journal.pone.0263885 PMC8830726

[52] Ahinkorah BO, Ameyaw EK, Seidu AA. Socio-economic and demographic predictors of unmet need for contraception among young women in sub-Saharan Africa: evidence from cross-sectional surveys. Reprod Health. 2020;17:1–1. 33097088 10.1186/s12978-020-01018-2 PMC7585192

[53] Angrist JD, Pischke JS. Mostly harmless econometrics: an empiricist’s companion. Princeton University Press; 2009.

[54] Strumpf EC, Harper S, Kaufman JS, Oakes M. Fixed effects and difference in differences. Methods Social Epidemiol. 2017;20:1.

[55] Wing C, Simon K, Bello-Gomez RA. Designing difference in difference studies: best practices for public health policy research. Annu Rev Public Health. 2018;39(1):453–69. 29328877 10.1146/annurev-publhealth-040617-013507

[56] ICF International. Survey organization manual for demographic and health surveys. MEASURE DHS. Calverton. Maryland: ICF International; 2012.

[57] De Chaisemartin C, d’Haultfoeuille X. Two-way fixed effects estimators with heterogeneous treatment effects. Am Econ Rev. 2020;110(9):2964–96.

[58] Sun L, Abraham S. Estimating dynamic treatment effects in event studies with heterogeneous treatment effects. J Econometrics. 2021;225(2):175–99.

[59] Goodman-Bacon A. Difference-in-differences with variation in treatment timing. J Econometr. 2021;225(2):254–77.

[60] Htun M, Jensenius FR. Expressive power of anti-violence legislation: changes in social norms on violence against women in Mexico. World Politics. 2022;74(1):1–36.

[61] Heymann J, Sprague A, Raub A. Equality within our lifetimes. University of California Press; 2023.

[62] Karra M. Measurement of unmet need for contraception: a counterfactual approach. Stud Fam Plann. 2022;53(4):657–80. 36495077 10.1111/sifp.12216 PMC10107817

[63] Moreau C, Shankar M, Helleringer S, Becker S. Measuring unmet need for contraception as a point prevalence. BMJ Glob Health. 2019;4(4): e001581. 31543991 10.1136/bmjgh-2019-001581 PMC6730575

[64] Sinai I, Igras S, Lundgren R. A practical alternative to calculating unmet need for family planning. Open Access J Contracept. 2017;26:53–9. 10.2147/OAJC.S137705 PMC5774555 29386953

[65] Jakiela P. Simple diagnostics for two-way fixed effects; 2021. arXiv:2103.13229.

[66] Callaway B, Sant’Anna PH. Difference-in-differences with multiple time periods. J Econ. 2021;225(2):200–30.

