# Supplementary material for: Domestic violence laws and women’s unmet need for family planning: Quasi-experimental evidence from Africa
Source: Reprod Health. 2025 Apr 26;22:60. doi: 10.1186/s12978-025-02011-3 (PMC12034134; doi:10.1186/s12978-025-02011-3)
Supplement: Supplementary file 1 — Additional file 1. [file 12978_2025_2011_MOESM1_ESM.docx]

Table A1: Detailed categories captured by the unmet need variable by DHS/IPUMS

| Variable categories | Value | All currently married or in-union women | All married or in-union women who have a demand for FP |
| --- | --- | --- | --- |
| Unmet need to space | 11 | 1 | 1 |
| Unmet need to limit | 12 | 1 | 1 |
| Using to space | 21 | 0 | 0 |
| Using to limit | 22 | 0 | 0 |
| Spacing failure | 31 | 0 | 0 |
| Limiting failure | 32 | 0 | 0 |
| No unmet need | 40 | 0 | Exclude |
| Desire birth in less than 2 years | 41 | 0 | Exclude |
| Infecund, menopausal | 51 | Exclude | Exclude |
| Never had sex | 52 | Exclude | Exclude |
| No sex, want to wait/not married | 53 | Exclude | Exclude |
| Unknown | 98 | Exclude | Exclude |
| Not in universe | 99 | Exclude | Exclude |
| Missing | . | Exclude | Exclude |
