# Supplementary material for: Domestic violence laws and women’s unmet need for family planning: Quasi-experimental evidence from Africa
Source: Reprod Health. 2025 Apr 26;22:60. doi: 10.1186/s12978-025-02011-3 (PMC12034134; doi:10.1186/s12978-025-02011-3)
Supplement: Supplementary file 2 — Additional file 2. [file 12978_2025_2011_MOESM2_ESM.docx]

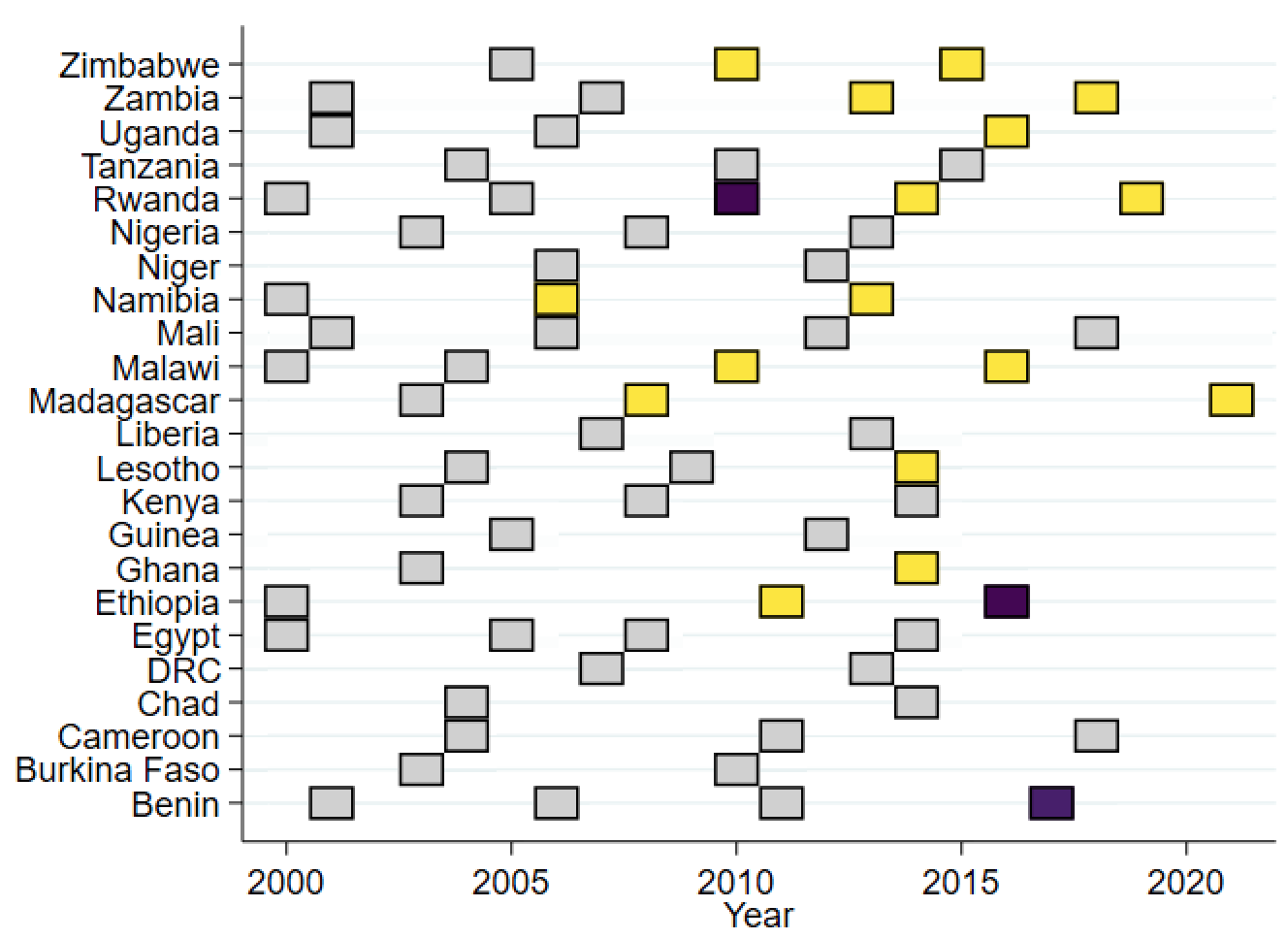


Figure A1: **Weights used for calculating the weighted average of the TWFE DD estimate.** Grey squares show untreated units, yellow squares show treated units with positive weight, and purple shows country-year combinations with negative weights. We excluded cases with negative weights that are marked in purple from the pooled analysis to assess the sensitivity of coefficient estimate to negative weights.
